# 30 years of primary health care reforms in Estonia: The role of financial incentives to achieve a multidisciplinary primary health care system

**DOI:** 10.1016/j.healthpol.2023.104710

**Published:** 2023-04

**Authors:** Triin Habicht, Kaija Kasekamp, Erin Webb

**Affiliations:** a WHO Barcelona Office for Health Systems Financing, Spain; b Institute of Family Medicine and Public Health, University of Tartu, Estonia; c Department of Healthcare Management, Berlin University of Technology, Germany; d European Observatory on Health Systems and Policies, Department of Healthcare Management, Berlin University of Technology, Germany

**Keywords:** Estonia, Primary health care, Multidisciplinary care, Health financing

## Abstract

Estonia has a legacy of hospital-focused service provision, but since the 1990s, has introduced a series of reforms to strengthen primary health care (PHC). The recent PHC reforms have placed an increasing focus on multidisciplinary care, involving home nurses, midwives, and physiotherapists, and emphasize PHC centres over single physician practices. These incremental reforms, without a supporting legal basis nor explicitly defined timelines and targets, nonetheless demonstrated the ability of financial incentives to drive change. EU structural funds in particular provided essential funding for infrastructure investments in PHC. Yet not all stakeholders supported these initiatives, largely due to the uncertain sustainability of funding. The EHIF also adjusted contract and payment terms to support PHC reforms, with some concessions to PHC providers operating as single practitioners. Despite substantial progress over the last three decades to shift the focus to PHC, there are some important bottlenecks that hinder the progress. These include PHC providers’ hesitance to give up their freedom as single practitioners, low interest from specialists to start working at the PHC level, and a lack of financial incentives and adequate funding for a broader scope of PHC services. This looks to become more challenging in the future, as nearly half of family physicians are 60 years old or older. The development of the new PHC strategy in 2023 is very timely to comprehensively address these bottlenecks and to set the vision for the future of PHC in Estonia.

## Highlights

• Strengthening primary health care (PHC) has been an Estonian reform priority.
• The PHC reforms emphasize multidisciplinary care and PHC centres over single providers.
• To encourage these changes, Estonia has introduced several financial incentives.
• EU structural funds have been extensively used to fund PHC infrastructure investments.
• Financial, legal, and workforce challenges to wider PHC reforms still remain.

## 1 Introduction

In the late eighties, similarly to other Eastern European countries, Estonia had an excessive hospital network and outpatient polyclinics. Local municipalities owned these facilities, which were staffed with various specialists [1]. These polyclinics carried out health prevention functions and included diagnostic units (e.g., laboratories, endoscopy), with the majority of the varied services provided by specialists [2]. The district doctors – the first contact of care – acted mostly as a gate keeper to specialists and provided a limited scope of services themselves [3].

In the early 1990s, Estonia began to rethink its health system approach and started to reorganize its primary health care (PHC) system. The reorganized PHC system aimed to centre around family doctors learning from other country experiences such as Finland, the UK and the Netherlands [4]. In 1991, Estonia developed retraining courses for working doctors and established family medicine as a clinical speciality. The major PHC reform started in 1998 when a critical mass of family doctors was trained and ready to work independently under new regulation and financial arrangements.

After the initial transformational reform started in 1998, Estonia continued to incrementally reform its PHC system. Estonia recognized the need to incentivize group practices to improve quality and increase efficiency already in 2003 [5]. Since 2009, the move towards multidisciplinary PHC has been one of the policy directions explicitly prioritized in policy documents. There is no single right way to assemble multidisciplinary PHC teams [6]. In Estonia, over time a series of workforce changes have been taking place accompanied by an important role of financial incentives designed to motivate PHC providers to take more responsibility for diagnostic services and treatment, to provide continuity of care, to compensate for the financial risks of caring for older people and to work in remote areas. These reforms have been accompanied by an increased understanding that small county hospitals should transform into person-centred community hospitals which are an essential part of integrated PHC network [7].

Such reforms, although incremental, are technically and politically complex. In this article we aim to review the evolution of the PHC reform and the role of health financing in moving towards multidisciplinary PHC in Estonia. We also provide an interpretation of Estonia's reforms in the context of international literature. This provides insights for policymakers in other counties designing and implementing reforms to strengthen PHC.

## 2 A series of policies and reforms related to multidisciplinary PHC

The move towards multidisciplinary PHC in Estonia has been incremental without explicitly defined timelines and targets. This section outlines the series of policies and reforms over time.

### 2.1 The first major reforms in the 1990s had a 5-year transition period to transform to family doctor-centred care

The PHC reform started in 1998 with a 5-year transition period to centre around a family doctor who meets most of the PHC needs of the population. The reform envisioned that the standard family physician practice should have a family doctor and a family nurse who work together, and individuals register with the family doctor under a practice list-system. The family doctor should be the coordinator of care and should operate as a gatekeeper referring her or his patients to higher levels of care when necessary [5]. Family physicians became private entrepreneurs or salaried employees of private companies owned by family doctors. In general, each family physician's practice list must contain between 1200 and 2000 patients, with an average practice list of around 1600 insured and 1700 total patients [8]. All together, these family physician practices cover the entire population.

One of the key features of the reform was the Estonian Health Insurance Fund's (EHIF) direct contract to PHC providers who were financially and legally separated from polyclinics. This separation prevented family physician practices from providing specialist care services [5]. In addition, a new funding model was developed. In 2000, there were 497 contracted PHC providers [9].

**Fig. 1:**
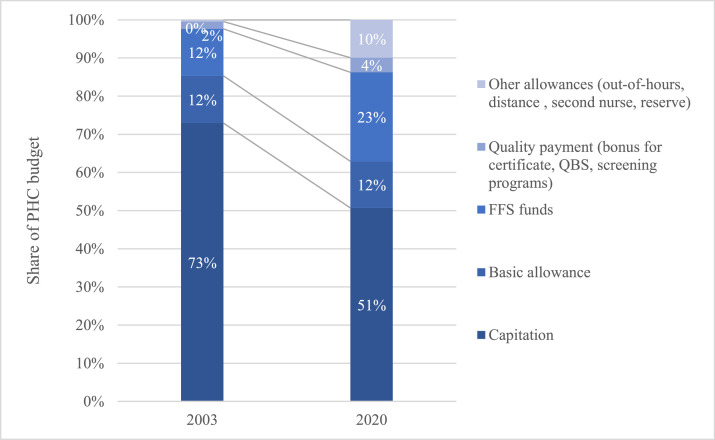
PHC payment design in 2003 and 2022.

Source: EHIF database.

Over time, the PHC budget has been increasingly transformed to a more blended payment model. The simple PHC payment system implemented in 1998 had five components: an age-weighted capitation fee per registered insured person per month, a fee-for-service fund, a basic monthly allowance, a distance fee, and a bonus for completing family medicine training. In addition to the five components outlined above by 2022, family doctors are also eligible for bonus payments including additional payments for quality, out-of-hour services, and expanding their team with e.g., a nurse or other experts. The transformation of the payment design is depicted in Fig. 1.

### 2.2 Incremental reforms have increased the role of family nurses within PHC teams over time

The duties of family nurses have increased substantially during the past two decades. As of 2022, nurses in Estonia can manage chronically ill patients, provide paediatric care, and even prescribe sick leave certificates and certain medications if they have completed required training. In 2012, the family nurse's role was regulated and compulsory individual visiting hours were implemented for nurses. In the early stages of the reform, the criteria that all family doctors with a patient list are required to work with at least one full-time family nurse was often not met [10]. Therefore, financial incentives were implemented and the providers with no full-time family nurse were paid 80% of the capitation. This requirement is still in place and in 2013, a separate allowance for the full-time second nurse per patient list was introduced. In 2020, there were only 9 patient lists that did not employ a full-time nurse at least once throughout the course of a full year and there were 594 patient lists in 2021 with a full-time second nurse, up from 182 in 2013 [11], [12], [13].

### 2.3 Subsequent PHC development plans introduced the PHC centre concept and expanded the PHC workforce

In 2009, the PHC development plan 2009–2015 was commissioned by the Ministry of Social Affairs (MoSA) [14]. For the first time, a PHC centre concept was introduced, mentioning physiotherapy and midwife services as a core part of PHC.

In 2014, the PHC centre approach was acknowledged and adopted in a political document describing Health System Development Plan up to 2020 (HSDP 2020) [15]. Compared to the previous development plan, the service package was extended to include home nursing. It defined that a PHC team serving a patient population of 5000–6000 should generally include at least 3–4 family doctors, 3–4 family nurses, a midwife, a physiotherapist, and a home nurse. PHC centres were also recommended to share infrastructure in locations with a nearby nursing hospital, ambulance, or specialist care provider.

### 2.4 Estonia used EU structural funds to invest in multidisciplinary PHC centres

**Fig. 2:**
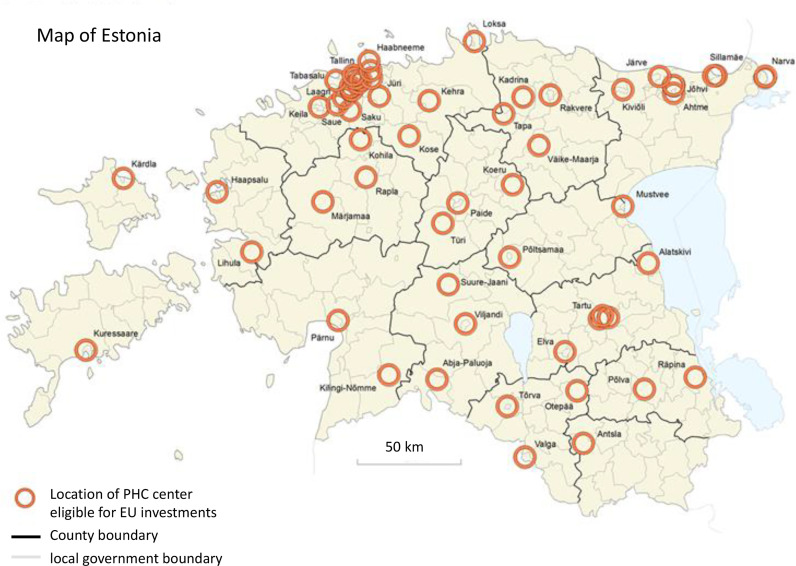
Locations of PHC centers eligible for EU investments.

Source: Estonian Statistics, Ministry of Social Affairs.

The HSDP 2020 was used as the basis to apply for the EU Structural Funds to invest in the infrastructure of PHC centres. The investments were very much needed, because PHC providers’ infrastructure was outdated and had become one of the key barriers in broadening the scope of PHC, moving to group practices, and building multidisciplinary teams. Eligibility for grants from the EU structural funds to construct or refurbish PHC centres required groups of at least three family doctors in rural areas or at least six family doctors in urban areas. Fig. 2 depicts the geographical locations for PHC centres eligible for EU funding. These locations were predefined by MoSA to assure that funding will go to centres of activity – where people actually live and use PHC services. MoSA used this approach to incentivize mergers of small, fragmented, and unsustainable practices. In addition to the geographical distribution of centres, locations of affiliate PHC were also predefined in smaller rural areas (not marked on the map) [16].

To promote the development of multidisciplinary teams, the centres eligible for the investment would be required to offer midwifery, home nursing and physiotherapy services. MoSA also promoted collaboration with other service providers like social services, pharmacy, dental care providers by allowing the use of EU funds to invest into their offices. The preparation application period for EU Structural Funds occurred from 2014, when the HDSP 2020 was adopted, until 2017. This was followed by the implementation phase from 2018 to 2023 [16].

### 2.5 EHIF introduced new contracts to support multidisciplinary PHC reforms

Parallel to the PHC development plans, in 2017, EHIF developed special contract terms and payment incentives for multidisciplinary PHC centers. The PHC centres are eligible for these additional incentives when at least three family doctors, with at least 4500 individuals on their patient lists, work together in one location. Moreover, they must have extended opening hours of at least 10 h per day, above the compulsory 8 h per day. If all conditions are met, then the PHC centre is eligible for the higher basic allowance [17]. The per capita payment is the same for all PHC providers. Following negotiations between PHC physicians and EHIF, it was also decided that EHIF could contract with multiple different legal entities for the PHC centre contract. The compromise was made because providers had low willingness to give up their own individually owned entities for group practices.

The PHC centres are obliged to provide midwife and physiotherapy services for at least 5 h per week and to conduct at least 18 home nurse visits during a half year period. Midwife services are covered on a fee-for-service (FFS) basis with no cap, including most essential laboratory tests. Physiotherapy services in PHC centres are also funded on a FFS basis with a cap of 10% from total capitation. PHC centres receive funding for home nurse services also on FFS basis without any cap and they are exempt from the open procurement process. Providers without PHC centre contracts have no obligation to provide these services. In the case of home nursing, providers would need to participate in open procurement process. PHC providers may choose to employ the necessary staff to provide these services or to contract with any other service provider [17].

In 2019, EHIF also introduced financing requirements for providers who are affiliated with a larger PHC centre but operate on separate premises and in different regions. In essence, a small, individual PHC provider (known as an affiliate practice) can start a cooperation with a larger PHC centre if they work in the larger PHC centre at least four hours per week. The affiliate practices are supported to improve services and ensure the sustainability of provision in rural areas. In 2021, 9 PHC centres had in total 15 affiliates, so the possibility of working as an affiliate is not widely used [18].

Starting from 2021, EHIF has implemented a new possibility of hiring additional staff in PHC centres. In these cases, EHIF would compensate the salary of these professionals based on their number of working hours at PHC centres. The providers may choose which expertise they want to hire amongst the choices of an extra nurse, a mental health nurse, a psychologist or a speech therapist [19]. By June 2021, only 10 PHC centres have used this option, for hiring 9 additional nurses and 1 mental health nurse [18]. The small uptake of this possibility could be explained by difficulties in motivating staff to work at PHC centres.

## 3 Stakeholder views about using EU structural funds

Although multidisciplinary PHC has been envisioned in multiple policy documents, the real trigger for change has been the access to EU funding. The initial political interest was to use this EU funding for specialist care infrastructure, but the EU agreed to provide the funding of 97.2 million only if Estonia used the funds on PHC service delivery reform, and not acute care or hospital infrastructure [20]. This funding put a strong pressure on MoSA to develop the criteria and principles for the EU grants which would also ensure changes in the service delivery model. The amount of funding the EU provided for infrastructure investments nearly reached the total EHIF funding for PHC of 103 million euros in 2016 [21].

**Fig. 3:**
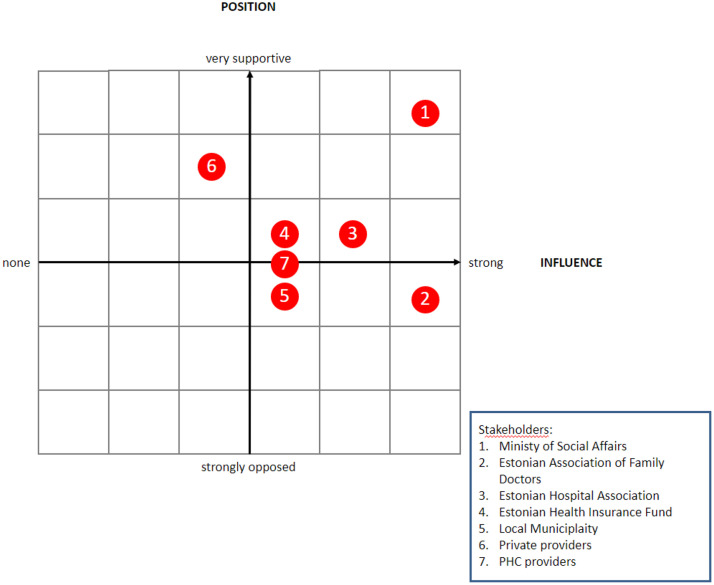
Stakeholder map: MoSA directed other stakeholders to apply for EU structural funds.

Source: Author's compilation.

MoSA saw the EU funding as an opportunity to incentivize smaller hospitals to focus more on PHC services. As such, MoSA allowed hospitals and local municipalities, in addition to PHC providers, to attain EU investments and build or refurbish infrastructure that they would rent out to PHC providers. This decision was heavily debated amongst different stakeholders taking different positions in the process (Fig. 3) and ultimately influenced the final financing criteria [16,22,23].

The Estonian Association of Family Doctors (EAFD) strongly opposed the initial proposal. The PHC providers expected that the state would undertake a bigger role in the process because PHC providers were not able to carry the financial risk and would have struggled to find funding for the cost sharing requirements of at least 25%. But strategically, MoSA used this opportunity to encourage and improve the often-missing dialogue between primary and secondary care The risk of losing the funding opportunity if the strict timeline for submitting the proposal was not met sped up the consensus-seeking process. In the end, the EAFD had high influence on the final eligibility criteria, although they had to accept that specialist care providers can apply for funding.

Views amongst PHC providers varied. Some of the PHC providers welcomed this long-awaited opportunity for funding to improve their infrastructure, but not all providers were ready for change. The PHC providers who had managed to build up their own independent successful practice were not keen to give up their independence and start working under someone else's influence or even on someone else's premises. As a compromise, PHC doctors were not mandated to change their legal status. Nevertheless, many PHC providers were still not convinced and therefore not all PHC providers benefited from EU funding. Therefore, only a little more than half (437 out of 799 family doctor lists at that point) signed commitments to start working in new facilities during the first round of applications in 2016 [24]. In following application round, the MoSA opened the application process only for locations which were not covered in the first round. The second application round ensured that at least one PHC centre was refurbished or built in each county [25].

EHIF had to navigate the expectations of PHC providers and MoSA, because there was no long-term commitment on additional resources for expanded PHC services. This created in some cases reluctance from PHC providers to apply for the EU funds, because there was no certainty on how the expanded services would be funded or how to pay for the rent on the new premises. When the PHC centres funding and contracting model was established in 2017, EHIF was in favour of allowing the increased funding only for the providers who merged as legal entities. However, MoSA strongly opposed this proposal, because it would mean that many of the facilities receiving EU funding may not be eligible for the PHC centre contract. Therefore, in practice PHC centre contracts are also signed with multiple legal entities of solo providers where EHIF often has to act as the steward to ensure smooth collaboration.

The Estonian Hospital Association was mostly supportive because part of the funding could also be used to reconstruct the existing ambulatory specialist care facilities. In addition, many hospitals saw the benefits of working more closely with the PHC providers. Renting rooms and providing additional services like physiotherapy, midwife and home nursing in collaboration with PHC providers would increase their funding and would help to mitigate the longer-term sustainability issues of smaller rural hospitals.

Private specialist and nursing care providers were supportive as they saw an opportunity to subsidize their infrastructure investments, as they were also eligible for EU funds. Initially, the EU regulation assumed that the providers applying for funding must ensure PHC service provision on the premises for five years. But MoSA extended the period to 20 years to mitigate the risk that private sector providers would refocus their use of infrastructure after the mandatory five-year period*.*

Local municipalities were also encouraged to apply for funds on behalf of the PHC providers. However, local municipalities do not have the responsibility to organize PHC and many of them were rather hesitant to take this long-term responsibility, despite MoSA's continuous efforts to increase their role.

## 4 Discussion and key lessons learned

Despite substantial progress over the last three decades to shift the focus to PHC, Estonia continues to face a tendency towards acute inpatient and outpatient specialist care located in hospitals [26]. The 2020 National Audit Office annual report focused on the future of essential public services (family doctors, teachers, police officers and rescuers). It concluded that PHC is the most problematic, as the number of permanently vacant positions has quadrupled in the last five years. Moreover, almost half of family doctors are 60 years of age or older [27].

The high share of solo practices has been highlighted as one of the main barriers to strengthening PHC [26]. As of October 2022, almost five years after introducing the new payment model for PHC centres, EHIF contracts with 60 PHC centres, merging 145 legal entities and comprising 340 family doctors, representing 43% of all patient lists in the country. These patient lists cover 44% of the registered population. Therefore, for 56%% of the Estonian population, the family doctors have no obligation to provide the extended PHC services to their enroled population [18,28,29]. This creates unequal access to services.

PHC providers that do not work under PHC centre contracts face many challenges. These challenges include the sustainability of the service due to the lack of substitutions, the lack of mentorship creating difficulties to achieve quality improvements and a big workload of managerial tasks. When the initial PHC model was developed, it was assumed that it would create incentives to merge into bigger group practices by saving costs on equipment and utilities [30]. However, no significant progress has been made even though the need to incentivize group practices was recognized to improve quality and increase efficiency already in 2003 [5]. At the time of the first reforms in the 1990s, the easiest form of entrepreneurship was self-employment. As a result, most of the PHC doctors chose to be self-employed, which resulted in individual management of their visiting hours and finances, allowing more freedom. This is one of the main barriers to developing multidisciplinary team-based care, because many providers are not willing to give up their freedom for working in a team of other PHC doctors. Another reason for the reluctance to switch to the PHC centre model may be that being a solo practitioner is more profitable, as a study conducted in 2015 indicated [30].

Financial incentives have always played an important role in PHC development. The main lesson learnt regarding the increasing the role of multidisciplinary PHC has been the importance of access to capital investments that enable and trigger the transformation of the model of care. PHC providers are small, risk-averse businesses in the Estonian context, which limits their willingness and ability to undertake necessary capital investments. At the same time, the lack of proper infrastructure has limited the ability of PHC providers to hire more staff and to move from solo to group practices. Lowered costs of capital investments have helped to overcome this barrier. A crucial role in this process is the strict requirement that capital investments should be accompanied by changes in the service delivery. The importance of the investments in infrastructure to ensure that PHC facilities can apply a multidisciplinary approach is fully aligned with international recommendations [6,31].

The second important lesson is that despite significant capital investments, having a lack of assurance and stability of funding for new service delivery models slows down the progress. During the EU funding application process, it was unclear how the costs for new services and even increasing costs for premises maintenance and rent would be covered. The funding model was only developed at the final stages of the application process. In addition, the funding did not give enough assurance that it would cover the costs of expanded services. Long term funding security in addition to capital investments is crucial to motivate changes in the model of care.

Third, this large-scale change in the service delivery model could have been better supported by revising the regulatory framework. In the Estonian case, no amendments were made to the Health Organization Act to formalize the increased scope of PHC. One of the reasons was the lack of broader consensus on the details of the new PHC model and it was easier to achieve the consensus on EU funding requirements. However, better aligned activities between MoSA and EHIF could have increased the speed and success of the reform, for example if MoSA had provided legal frameworks for multidisciplinary PHC and EHIF had assured sustainable financing.

Fourth, the contracting terms and payment incentives could be used to motivate changes in service delivery models [32]. In the Estonian context, the existing PHC centre payment design may not be enough to incentivize bigger changes in the service delivery model. The only financial incentive is the higher level of basic allowance for PHC centers, but this incentive aims to motivate providers to sign the PHC centre contract, rather than specifically incentivizing providers to increase and broaden their scope of PHC services. Payment rates for midwife, physiotherapy and home nursing services are the same for providers working under the PHC centre contracts and for other PHC providers. While there have been incremental changes to the contracts, funding model, and payment design with PHC centres, these have not been sufficient and aligned to see a recognizable shift toward multidisciplinary teams at the PHC level.

Fifth, financial incentives and amending regulation alone are not enough – moving from solo practices to a teamwork and multidisciplinary-based approach requires much more*.* The legacy of PHC provision in Estonia still limits the possibilities for PHC reforms. When the PHC system was initially implemented, there was much focus on separating PHC providers from hospitals and even working as solo entrepreneurs. This persists in the memory of PHC providers to whom the reform may have been extremely challenging. Therefore, there is still resistance to any change, especially one that seems like a step back towards the direction of working again more closely with hospitals. In addition, the average age of family physicians may limit any significant service delivery or organizational developments at the PHC level, as providers planning to retire soon may have low willingness to change.

Lastly, one success factor of the reform was the careful stakeholder engagement. MoSA was able to find a balance between different interests by ensuring that the reform package offers something for different stakeholders. MoSA's approach of providing each stakeholder with the possibility to participate and receive a financial incentive ensured vast interest in the reform. Nevertheless, MoSA managed the interests of different stakeholders by very strictly regulating the obligations that accompany the funds.

## 5 Conclusion

Despite efforts, progress towards multidisciplinary PHC has been slower than anticipated. Although the EU investment support and new EHIF financial incentives have played a crucial role in moving towards multidisciplinary PHC, there are some important bottlenecks that hinder the progress. These include challenges in implementing a new legislative basis, PHC providers’ hesitance to give up their freedom as single practitioners, a lack of interest from specialists to start working at the PHC level, and a lack of financial incentives and adequate funding for a broader scope of PHC services. The next steps should concentrate on eliminating possible bottle necks by closely monitoring the progress and making necessary adjustments in PHC funding and regulation. The development of Estonia's new PHC strategy by 2023 provides a great opportunity to evaluate what has worked with the PHC reforms so far and what can be done in future reforms to enable the desired changes. Whilst this paper focuses on the Estonian PHC reforms, other countries may face similar bottlenecks and policy choices, especially given the increasing need for multidisciplinary care.

## Disclaimer

The authors alone are responsible for the views expressed in this publication and they do not necessarily represent the decisions or policies of the World Health Organization.

## Funding

This research did not receive any specific grant from funding agencies in the public, commercial, or not-for-profit sectors.

## Declaration of Competing Interest

We declare no competing interests.
